# Short Editorial - Effect of Passive Smoking on Blood Pressure Response to Epinephrine and Felypressin in 1K1C Hypertensive Rats Treated or not with Atenolol

**DOI:** 10.36660/abc.20200032

**Published:** 2020-02

**Authors:** Paulo J. F. Tucci

**Affiliations:** Universidade Federal de São Paulo (UNIFESP), São Paulo, SP - Brazil

**Keywords:** Rats, Tobacco Use Disorder, Tobacco/adverse effects, Anesthesia, Dental, Epinephrine, Felipressin, Hypertension, Atenolol

I begin this text highlighting the importance of multidisciplinarity in the development of knowledge.

In 1842, Johann Christian Doppler, an Austrian physicist, defined that the frequency of the star’s light changed with its motion, as it moved closer or away. One and a half century later, such “useless curiosity” started to be applied for the diagnosis of congenital heart diseases. And for a long time, we do know when the train is coming or going.

Needless to highlight the similarities among the areas of biological sciences, with emphasis on animal experiments. Affinities with Cardiology are described in this publication.^1^

Felypressin is an analogue of vasopressin (also called antidiuretic hormone or ADH), a hormone produced in the neurohypophysis. In Dentistry, felypressin is of special interest because it acts as a vasoconstrictor that prolongs the anesthetic effect.

In an artificially induced hypertensive model (and using a protocol illustrated in Figure 1 of the commented text), the effect of atenolol and felypressin on blood pressure was assessed in artificially hypertensive rats submitted to anesthesia, exposed to cigarette smoke. The results indicated that smoking can reduce epinephrine-induced vasodilation and increase the hypertensive response compared with felypressin.

These data add to the literature on the cardiac effects of vasoconstrictors. A recent review,^2^ however, concluded that more studies are needed to increase the strength of the evidence.

It is interesting to note the elevated blood pressure of the anesthetized rats. Systemic arterial hypertension is not a common response to ketamine and xylazine,^3^ suggesting that the dose of anesthetic was not sufficient to prevent the increase in sympathetic activity in the operated rats.

A recent study with awake humans, published in Arquivos Brasileiros de Cardiologia,^4^ concluded that “felypressin increased the diastolic blood pressure of hypertensive patients with controlled blood pressure. Patients with high trait anxiety presented increases in systolic blood pressure upon some procedures”. It is worth pointing out that the arterial catheter was placed after occlusion of the left carotid artery, which may have activated baroreceptors located in the carotid sinus, resulting in arterial hypertension. Maybe femoral artery catheterization would have been better.

Also, the time of cigarette smoke exposure was short (10 minutes/day), different from other studies in which the animals are exposed for longer periods.^5^

The publication of this study in the Arquivos Brasileiros de Cardiologia is timely; many heart disease patients want to know about the risks involved in their dental treatment, and physicians should be prepared for it.
